# A Novel Herbal Extract Blend Product Prevents Particulate Matters-Induced Inflammation by Improving Gut Microbiota and Maintaining the Integrity of the Intestinal Barrier

**DOI:** 10.3390/nu14102010

**Published:** 2022-05-11

**Authors:** Lilan Jin, Lu Deng, Mark Bartlett, Yiping Ren, Jihong Lu, Qian Chen, Yixiao Pan, Hai Wang, Xiaokui Guo, Chang Liu

**Affiliations:** 1 Department of Immunology and Microbiology, Shanghai Jiao Tong University School of Medicine, Shanghai 200025, China; jll40690@rjh.com.cn (L.J.); 18217721131@163.com (L.D.); 517710910010@shsmu.edu.cn (Y.P.); 517710910026@shsmu.edu.cn (H.W.); 2 Department of Clinical Laboratory Medicine, Ruijin Hospital Affiliated to Shanghai Jiao Tong University School of Medicine, Shanghai 200025, China; 3 Center for Anti-Aging Research, Nu Skin Enterprises, Provo, UT 84601, USA; mrbartle@nuskin.com; 4 Nu Skin (China) Daily-Use & Health Products Co., Ltd., Center of Anti-Aging Research, Nu Skin Enterprises, Shanghai 201401, China; yipingren@nuskin.com (Y.R.); jihonglu@nuskin.com (J.L.); 5 Institute of Intestinal Diseases, Shanghai Tenth People’s Hospital Affiliated to Tongji University, Shanghai 200072, China; chenmengze@126.com; 6 School of Global Health, Chinese Center for Tropical Diseases Research, Shanghai Jiao Tong University School of Medicine, Shanghai 200025, China; xkguo@shsmu.edu.cn

**Keywords:** PM2.5, inflammation, gut microbiota, herbal extract blend

## Abstract

Air pollutants of PM2.5 can alter the composition of gut microbiota and lead to inflammation in the lung and gastrointestinal tract. The aim of this study was to evaluate the protective effect of a novel herbal extract blend, FC, composed of Lonicera japonica extract, Momordica grosvenori extract, and broccoli seed extract, on PM2.5-induced inflammation in the respiratory and intestinal tract. A549 cells and THP-1 cells, as well as C57BL/6 mice, were stimulated with PM2.5 to establish in vitro and in vivo exposure models. The models were treated with or without FC. The expression of inflammatory cytokines and tight junction proteins were studied. Proteomic analysis was performed to elucidate mechanisms. Mouse feces were collected for gut microbiota analysis. FC was shown to modulate the upregulation of pro-inflammatory cytokines mRNA expression in A549 and THP-1 cells and downregulated tight junction proteins mRNA expression in A549 cells due to PM2.5 stimulation. In animal models, the decreased expression of the anti-inflammatory factor *il-10*, tight junction protein ZO-1, and the elevated expression of COX-2 induced by PM2.5 were improved by FC intervention, which may be associated with *zo-1* and *cox-2* signaling pathways. In addition, FC was shown to improve the gut microbiota by increasing the abundance of beneficial bacteria.

## 1. Introduction

The impact of air pollution on public health continues to be a focus of attention around the world. While previous studies on air pollution primarily focused on the respiratory and cardiovascular effects, emerging evidence supports a significant impact of air pollution on the gastrointestinal (GI) system. Air pollutants include gaseous pollutants and particulate matters (PM). PM2.5 (particles less than 2.5 μm in diameter) have been shown to be an important airborne pathogenic factor. PM2.5 has a large surface area and can adsorb a variety of toxic and harmful substances [1], which can penetrate deeply into the lung and deposit in the terminal bronchioles and alveoli, irritate and corrode the alveolar wall, and consequently impair lung function. An increase of 10 μg/m^3^ per day of PM2.5 concentration increases 0.29% of overall non-accidental mortality and 0.22% of respiratory disease mortalities [2]. It has been widely reported that PM2.5 stimulated the overexpression of a number of inflammation-related cytokine genes, which can induce eosinophil, neutrophil, and T cell migration to the lung, the intestine, and other tissues [3,4]. Additionally, human alveolar macrophages treated with PM2.5 express high levels of M1-associated proinflammatory cytokines (IL-12 and IFN-γ) and low levels of M2-associated anti-inflammatory cytokines (IL-4, IL-10, and IL-13) [5,6,7]. Recent studies showed that PM2.5 induces an inflammatory response in macrophages via activation of TLR4/NF-κB/COX-2 signaling [8]. Cyclooxygenase-2 (COX-2), a key enzyme in fatty acid metabolism, is upregulated during both inflammation and cancer [9]. COX-2 is expressed in response to inflammatory factors, other physiological stimuli, and growth factors, and is involved in the production of those prostaglandins that mediate pain and support the inflammatory process [10]. The inflammatory factors induced by PM2.5 can synergistically act to damage lung cells.

PM2.5 can also enter the intestinal tract, mainly through mucociliary clearance and oral intake [11]. PM2.5 has been shown to increase intestinal epithelial permeability and impair intestinal immune barrier function, and can also be metabolized into toxic byproducts, further causing intestinal injury [4,12]. Intestinal epithelial permeability is partly determined by tight junction protein expression. Among the tight junction-associated proteins studied, ZO-1 has garnered particular attention in recent years. ZO-1 is a junctional adaptor protein that interacts with multiple other junctional components. It is an essential orchestrator of endothelial spatial actomyosin organization, and thereby regulates tensile force acting on adherens junctions, recruitment of tight junction proteins, and endothelial cell migration [13]. The levels of paracellular barrier and actin reorganization are decreased in ZO-1 knockdown cells [14]. The transcript and protein levels of tight junction markers, E-cadherin, ZO-1, and occludin, were found to decrease after PM2.5 exposure in vivo and in vitro [15]. ZO-1 is also found to be dispensable for barrier function but critical for effective mucosal repair [16].

An ecologic analysis study demonstrated that total emissions of six documented pollutants, including PM2.5, were directly correlated with the county-level rate of IBD (inflammatory bowel disease)-related hospitalizations and that every log increase of pollutants was linked to a 40% increase in hospitalizations for both UC (ulcerative colitis) and CD (Crohn’s disease) [17]. Few studies existed elucidating the immune mechanisms of PM2.5 triggering an inflammatory reaction in the gut. However, one study showed that exposure to PM2.5 induced elevated TNF-α expression only in the colon and did not affect the expression of other cytokines [11].

In recent years, advances in culture-independent DNA sequencing of the human microbiome have shed light on the importance of microbe-host interactions that are reshaping our understanding of human disease and toxicology. Some studies have shown that PM2.5 can alter the composition of the gut microbiome, which is thought to be closely related to the functioning of multiple systems in the body [11,18]. In addition, emerging evidence illustrates that the airway, even in its healthy state, is not sterile. Bacteria enter the lungs by direct mucosal dispersion and micro-aspiration from the URT [19], as well as by direct inhalation of ambient air. 16S rRNA gene sequencing indicated that PM2.5 exposure significantly altered the richness, evenness, and composition of the lung microbiome [20]. Current studies have shown that any imbalance of the lung microbiome is closely related to the development of a variety of diseases, including pulmonary cystic fibrosis, COPD, asthma, and lung cancer [21,22,23,24].

Due to the multi-system influence of PM2.5, it is difficult to design meaningful regimens to decrease the risks of lung pathological changes. Herbal extracts provide new insights for designing therapeutic agents, considering their multiple therapeutic effects on the human body. For this study, three natural extracts were selected as adjuvant therapy, including Lonicera japonica, Momordica grosvenori, and broccoli seed extract. These three natural extracts have previously been shown to have anti-inflammatory, antioxidant and antitumor effects [25,26,27,28,29,30,31,32]. Both Lonicera japonica and broccoli seed extract have antivirus effects [33,34], with the former also demonstrating antibacterial effects [35]. Specifically, Lonicera japonica and broccoli seed extract have been shown to provide beneficial effects on intestinal homeostasis, although there are very few reports on the effect of Momordica grosvenori on the intestinal tract. For example, a water extract of Lonicera japonica showed protective effects against DSS-induced colitis via the Th1/Th17 pathway [36]. Another study showed that mdr1a (-/-) mice fed with a broccoli-supplemented diet significantly lowered colonic inflammation, compared to the control diet [37]. It is reasonable to suppose that an herbal extract blend containing these three components could prevent PM2.5-induced intestinal inflammation and modulate the balance of human microbiota.

This study constitutes the investigation of an herbal extract blend named “Fresh Clear” (FC), composed of Lonicera japonica extract, Momordica grosvenori extract, and broccoli seed extract. The recommended adult dose of FC is 2900 mg/kg/day, including Lonicera japonica extract 500 mg/kg/day [38,39], Momordica grosvenori extract 50 mg/kg/day [40], broccoli seed extract 125 mg/kg/day [29], with the balance being excipients. We designed this study to verify whether FC could be effective in suppressing inflammation and addressing the disturbance of gut microbiome caused by PM2.5. The study provides a basis for the further application of this herbal extract blend.

## 2. Materials and Methods

### 2.1. Collection and Composition Analysis of PM2.5

PM2.5 was collected from 7 September 2020 to 12 January 2021 in Shanghai, using type XA 1002 high volume PM2.5 sampler (QingDao qdxao Environmental Technology Co., Ltd., Qingdao, China) at a flow rate of 1.050 m^3^/min. The collected PM2.5 samples were stored in a −80 °C refrigerator. As shown in previous studies, the components of PM2.5 include metals, sulfides, and polycyclic aromatic hydrocarbons (PAHs). Inductively coupled plasma optical emission spectrometry was used to detect the ion content in the samples. Methylene blue spectrophotometry was applied in a sulfide test and gas chromatography/mass spectrometry was used to detect PAHs. 

### 2.2. The Herbal Extract Blend Fresh Clear (FC)

The FC (supplied by the Center for Anti-aging Research, Nu Skin Enterprises, Shanghai, China) was composed of monk fruit (Luo Han Guo) extract (Mogrosides V ≥ 55%), honeysuckle (Lonicera japonica) extract (chlorogenic acid ≥ 3.5%), and broccoli seed extract (glucoraphanin ≥ 13.0%) in the form of freeze-dried powder. The powder was suspended in saline before treatment.

### 2.3. Cell Culture and Cytotoxicity Test

Since, as previously described, PM2.5 can enter the respiratory tract and impact the development of respiratory diseases and multi-system inflammation, we selected a human lung epithelial cell line and an immune cell line for the PM2.5 stimulation experiments. A549 cells are a well-characterized standard among the human lung carcinoma/alveolar cell lines used in molecular biology [41]. The THP-1 cell line has been widely used to study immune responses while cells are not only in the monocyte state but also in the macrophage-like state [42]. We studied FC in cells to determine its impact on inflammatory factors induced by PM2.5 and its impact on the maintenance of gut barrier integrity.

A549 cells were cultured in high glucose DMEM medium supplemented with 10% fetal bovine serum and 100 Units/mL streptomycin and penicillin, maintained at 37 °C in a 5% CO_2_ incubator. THP-1 cells were cultured in RPMI 1640 medium supplemented with 10% FBS and 100 units/mL streptomycin and penicillin, maintained at 37 °C in a 5% CO_2_ incubator.

The Cell Counting Kit-8 assay, referred to as CCK-8 assay or CCK8 assay, provides a convenient and robust way of performing a cell viability assay. The kit uses a water-soluble tetrazolium salt to quantify the number of live cells by producing an orange formazan dye upon bio-reduction in the presence of an electron carrier. For the same cells, there is a linear relationship between the shade of color and the number of cells.

A CCK8 assay was conducted, following the instructions, to estimate the highest possible concentration of herbal extract blend (FC) that is non-toxic to the cells. An FC concentration with an OD value greater than or equal to the OD value of control cells without FC pretreatment was considered non-toxic to the cells. Approximately 4 × 10^4^ cells were seeded in 96-well plates with 100 μL culture medium in each well. After 24 h of incubation, the medium was replaced by serum-free DMEM. After 12 h of cell culture, the serum-free medium was replaced with the complete medium, with or without FC. The concentrations of FC tested were 10 mg/mL, 5 mg/mL, 1 mg/mL, 0.1 mg/mL, and 0.01 mg/mL. All experiments were conducted in five replicates. A 10 μL CCK8 solution was added to each well and incubated in the dark for 0 h, 0.5 h, 1 h, 2 h, 6 h, and 12 h. The absorbance values were measured at 450 nm and 630 nm. OD = A450 nm–A630 nm. The control group included cells without FC treatment, and the FC solutions without cells were considered as blank, so that the OD values could be subtracted.

### 2.4. Cell Experiment

About 4 × 10^5^ A549 and THP-1 cells were seeded in 12-well plates with 1 mL of medium per well. After 24 h of incubation, the complete medium was exchanged for serum-free medium. After 12 h, the culture medium was replaced with the complete medium with 0.02 mg/mL PM2.5, and/or with 1 mg/mL FC. Medium without FC was used as a control. The PM2.5 doses were determined based on previous studies [43,44]. Cells were collected after stimulation for 0.5 h, 1 h, 2 h, and 6 h. All the experiments were repeated in triplicate. The total RNA of the cells was then extracted. The expression of target genes (*il-1β, il-6, il-8, tnf-α, ifn-γ, cox-2, claudin-1 and zo-1*) was analyzed with quantitative real-time PCR with the 2^−ΔΔCT^ method. The primer sequences are shown in Table 1.

### 2.5. Animal Experiment

Sixty male C57BL/6 mice (4–6 weeks old, 16–20 g, specific pathogen free) were maintained at the animal laboratory center of the Shanghai Jiao Tong University School of Medicine. The experimental procedure was approved by the Animal Welfare Committee of the Shanghai Jiao Tong University School of Medicine. The mice were allowed to acclimate for 1 week in a controlled environment (temperature 20 ± 2 °C and humidity 55 ± 5% with a 12-h/12-h light/dark cycle). Then, the mice were randomly assigned to four groups (15 mice respectively in three cages for the groups of control, PM, PM + FC, and FC). For group PM + FC and group FC, the mice were administered with 580 mg/kg/day FC by gavage during the following 13 weeks, while for group PM and the control group (group C), the mice were administered with saline by gavage. The dosage of FC for mice = 2900 mg/day (recommended dosage for human, excipients included) ÷ 60 kg × 12 (the dosage conversion ratio of mouse and human). The recommended dose for humans is based on data in the literature [29,38,39,40]. The mice in group PM + FC and group PM were subjected to 10 μg/g body weight of PM2.5 [45] (dissolved in 45 μL of saline) via nasal drip and to the same amount of PM 2.5 (dissolved in 200 μL of FC solution and saline, respectively) by gavage on alternate days during the 15th and 16th weeks, while group FC and group C were subjected to saline alone via nasal drip and to 200 μL of FC solution and saline, respectively, by gavage. Body weight was monitored every week. Stool samples were collected before FC gavage (T1), before nasal drip (T2), and after nasal drip (T3). After 16 weeks’ treatment, all of the mice were humanely sacrificed. Lung samples and intestinal samples were collected. Two pieces of lung samples and two pieces of 1.5 cm long samples of intestinal tissue were immediately preserved in liquid nitrogen and stored in a −80 °C refrigerator for subsequent RNA extraction and protein assay.

### 2.6. Inflammatory Factors and Tight Junction Proteins Detection of Animal Tissues

The total RNA of lung and colon tissues of each mouse was extracted using TRIzol, following the instructions. The transcript levels of *il-10*, TNF-α, COX-2, and ZO-1 were determined by real-time quantitative PCR after reverse transcription. The results were calculated according to the 2^−ΔΔCT^ method. The primer sequences are shown in Table 1. The animal tissues were extracted, and the total protein concentration was determined with a Pierce™ BCA Protein Assay Kit. The expression of TNF-α, COX-2, and tight junction protein ZO-1 were detected by Western blot. β-ACTIN was used as an internal reference. Protein bands were developed by ImageQuant LAS-4000 MINI. Quantity One was used for the grayscale scanning of strips.

### 2.7. Histological Analysis

Lung and colon tissues fixed in paraformaldehyde were embedded in paraffin and cut into 5-µm-thick sections. To deparaffinize, the sections were immersed in xylene at 56 °C twice for 20 min, and hydrated with ethanol (twice with 100%, once with 95%, and once with 75% ethanol) for 5 min. The tissues were processed routinely for hematoxylin and eosin (H&E) histology.

### 2.8. Proteomics Analysis

Preparations of 100 µg protein from mouse lung tissues were made in triplicate for each group. For each sample, proteins were precipitated with ice-cold acetone, and then re-dissolved in 100 µL TEAB (tetraethyl-ammonium bromide). Proteins then underwent digestion with 1:50 sequence-grade modified trypsin, and the resultant peptide mixture was labeled with iTRAQ (isobaric tags for relative and absolute quantitation). The peptide mixture was separated at high pH; twelve fractions were collected, then each fraction was dried in a vacuum concentrator in preparation for the next step. Then, nano-HPLC (high-performance liquid chromatography)-MS (mass spectroscopy)/MS analysis was conducted at low pH. Scaffold (version Scaffold_4.7.5, Proteome Software Inc., Portland, OR, USA) was used to validate MS/MS-based peptide and protein identifications. Scaffold Q+ (version Scaffold_4.7.5, Proteome Software Inc.) was used to quantitate peptide and protein identifications. QPCR and Western blot were used to detect the levels of any functional proteins found in mouse lung tissues.

### 2.9. Fecal Microbiota Analysis

Total DNA was extracted from fecal samples and 16S rDNA high-throughput sequencing was performed by the Realbio Genomics Institute (Shanghai, China) using the Illumina HiSeq PE250. Variable regions V3–V4 on 16S rDNA genes of bacteria were amplified with forward primer F3415′-ACTCCTACGGGRSGCAGCAG-3′ and reverse primer R806 5′-GGACTACVVGGGTATCTAATC-3′. The raw paired end reads were assembled by pandaseq with overlap nucleotides. The reads were then quality-filtered. The raw data were then subjected to a quality control procedure using UPARSE. The qualified reads were clustered to generate operational taxonomic units (OTUs) at the 97% similarity level using Usearch, where a sequence is selected from each OTU as the representative sequence of the OTU. This representative sequence was compared with the 16S database of known species (RDP, http://rdp.cme.msu.edu, accessed on 11 February 2020) using the RDP method, in order to classify each OTU as a species. After classification, an OTU abundance table was obtained according to the number of sequences in each OTU. According to species annotation, the relative abundance of each sample annotated to the taxonomic level (kingdom, phylum, class, order, family, genus) was calculated. At the phylum, class, order, family, and genus levels, the sequence numbers of each annotated species or the OTU in different samples were organized into a table to form a profiling histogram. Heatmap analysis and the difference analysis among four groups were performed using R. QIIME software was used to calculate the α diversity index and the β diversity index of the samples. The rank sum test was used to analyze the significant differences between different groups to determine the species that had a significant difference between the groups.

### 2.10. Statistical Analysis

All statistical analysis was performed using GraphPad Prism 7.0 software. All data were reported as mean ± SEM. Statistically significant differences among groups were identified according to 2-way analysis of variance (ANOVA) followed by Tukey’s multiple comparisons test when the data conformed to a normal distribution and Kruskal Wallis test followed by Dunn’s multiple comparisons test when the data did not fit a normal distribution. Results were considered statistically significant at * p < 0.05, ** *p* < 0.01, *** *p* < 0.001, **** *p* < 0.0001.

## 3. Results

### 3.1. The Components of PM2.5

The concentrations of these components in the collected PM2.5 samples are listed in Table 2. According to Ambient Air Quality Standards of the national standards of the People’s Republic of China (GB 3095-2012), the reference value of sulfur dioxide and benzo(a)pyrene concentrations are respectively 60 μg/m³ and 0.001 μg/m³, which are far above the sulfide and benzo(a)pyrene concentrations in the PM2.5 we collected in Shanghai.

### 3.2. Cell Proliferation Treated by Herbal Extract Blend (FC)

**Figure 1:**
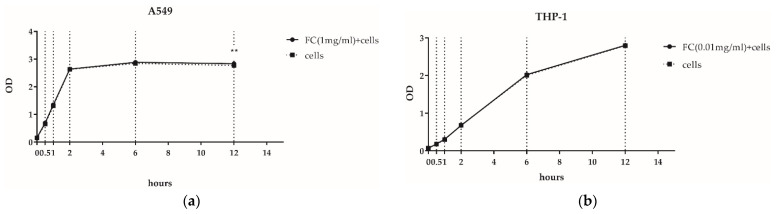
Cell proliferation assays with the CCK8 method in 12 h. (The error bars are shorter than the height of the dots here.) (**a**) A549 cells; (**b**) THP-1 cells. ** *p* < 0.01.

The CCK8 assays showed that the highest FC concentration that is non-toxic to A549 is 1 mg/mL (Figure 1a), while the highest FC concentration that is non-toxic to THP-1 is 0.01 mg/mL (Figure 1b). Therefore, 1 mg/ mL was used as the FC concentration in the subsequent A549 experiment, and 0.01 mg/ mL was used as the FC concentration in the THP-1 experiment.

### 3.3. Herbal Extract Blend (FC) Downregulates the Expression of Inflammatory Cytokines in Cells Induced by PM2.5

**Figure 2:**
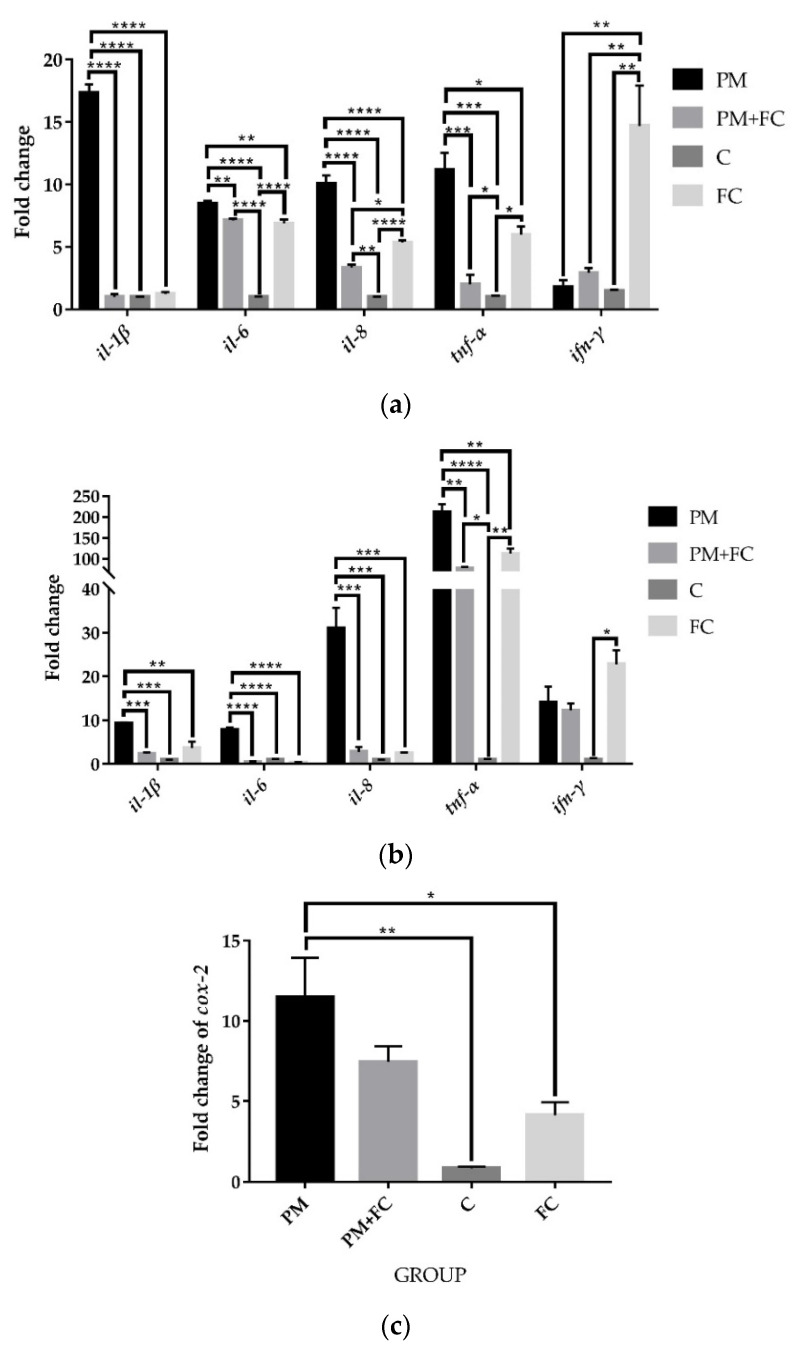
Fold changes of inflammatory cytokines in cells: (**a**) fold changes of inflammatory cytokines in A549 cells after treatment for 6 h; (**b**) fold changes of inflammatory cytokines in THP-1 cells after treatment for 2 h; (**c**) fold change of *cox-2* in A549 cells after treatment for 2 h. * *p* < 0.05, ** *p* < 0.01, *** *p* < 0.001, **** *p* < 0.0001.

After treatment with different groups, including PM2.5 (group PM), PM2.5 + herbal extract blend FC (group PM + FC), FC (group FC), or medium only (group C), for 6 h, the gene expressions of *il-1β*, *il-8*, and *tnf-α* in group PM were significantly upregulated, while the results of group PM + FC and group FC were found to be significantly lower than those in group PM (Figure 2a). After the same stimulation in THP-1 cells, similar results were observed (Figure 2b). The fold change of *cox-2* in group FC was also found to be significantly lower than those in group PM in A549 cells after treatment for 2 h (Figure 2c). Although no significant differences between group PM and group PM + FC in *cox-2* expression were found, a downward trend in group PM + FC was still observed. These results indicate that the herbal extract blend (FC) can downregulate the expression of inflammatory cytokines at the cell level. 

### 3.4. Tight Junction Protein Expression in A549 Cells

**Figure 3:**
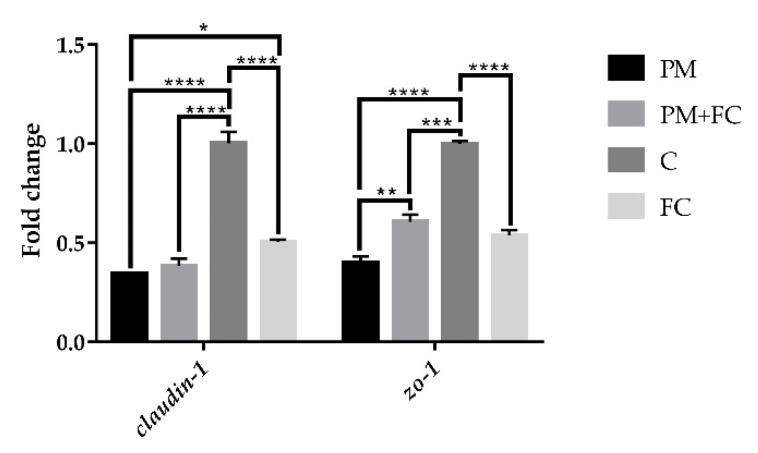
Fold changes of tight junction proteins in A549 cells after stimulating for 6 h. * *p* < 0.05, ** *p* < 0.01, *** *p* < 0.001, **** *p* < 0.0001.

In order to verify the effect of FC on the intestinal epithelial barrier, we also measured mRNA expression of tight junction proteins in A549 cells. As shown in Figure 3, after stimulation for 6 h, the mRNA expression of *claudin-1* was significantly downregulated in group PM compared with group C, while in group FC, the mRNA expression of *claudin-1* was higher than it was in group PM. However, there was no significant difference between group PM + FC and group PM. For *zo-1*, the mRNA expression in group PM was lower than that of group C, and FC upregulated the expression reduced by PM in group PM + FC in A549 cells.

### 3.5. The Anti-Inflammatory Effects of Herbal Extract Blend (FC) in Animal Tissues

**Figure 4:**
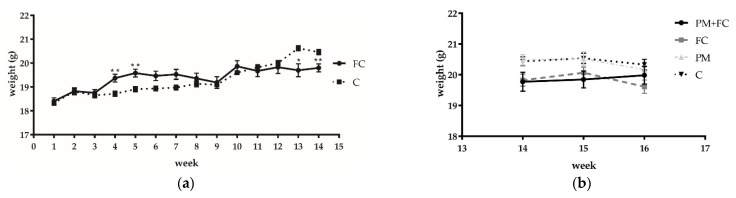
Mice weight monitoring data of each week before (**a**) and after (**b**) PM2.5 nasal drip experiment. * *p* < 0.05, ** *p* < 0.01.

**Figure 5:**
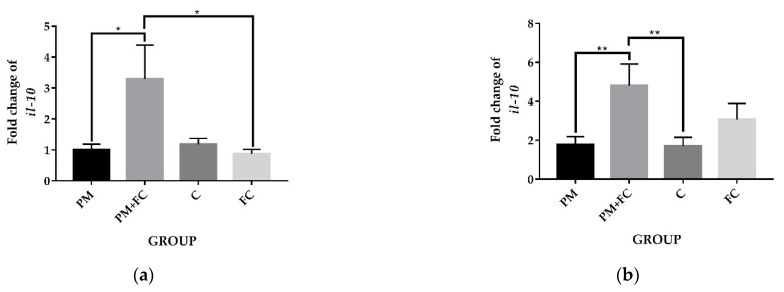
Fold changes of mRNA expression of *il-10* in animal tissues.:(**a**) mouse lung tissues; (**b**) mouse colon tissues. * *p* < 0.05, ** *p* < 0.01.

Mice pretreated with FC (group FC) had significantly higher body weight than mice pretreated with saline (group C) in the 4th and 5th weeks. After the 5th week, the body weights of the mice in group FC and the mice in group C tended to be the same, and by the 13th and 14th weeks, the weight of the mice in group FC was significantly lower than that of the mice in group C. However, at the 15th and 16th weeks, no significant differences were observed among different groups (Figure 4). Moreover, no significant inflammatory infiltration was found in the tissues. The mRNA expression of *tnf-α* and other inflammatory factors were not detected in the tissue histopathological tests. However, as can be seen from Figure 5, the expression levels of *il-10* in group PM + FC were significantly higher than the level in group PM.

### 3.6. Modulation of Herbal Extract Blend (FC) on ZO-1 and COX-2 Expression in Animal Tissues

**Figure 6:**
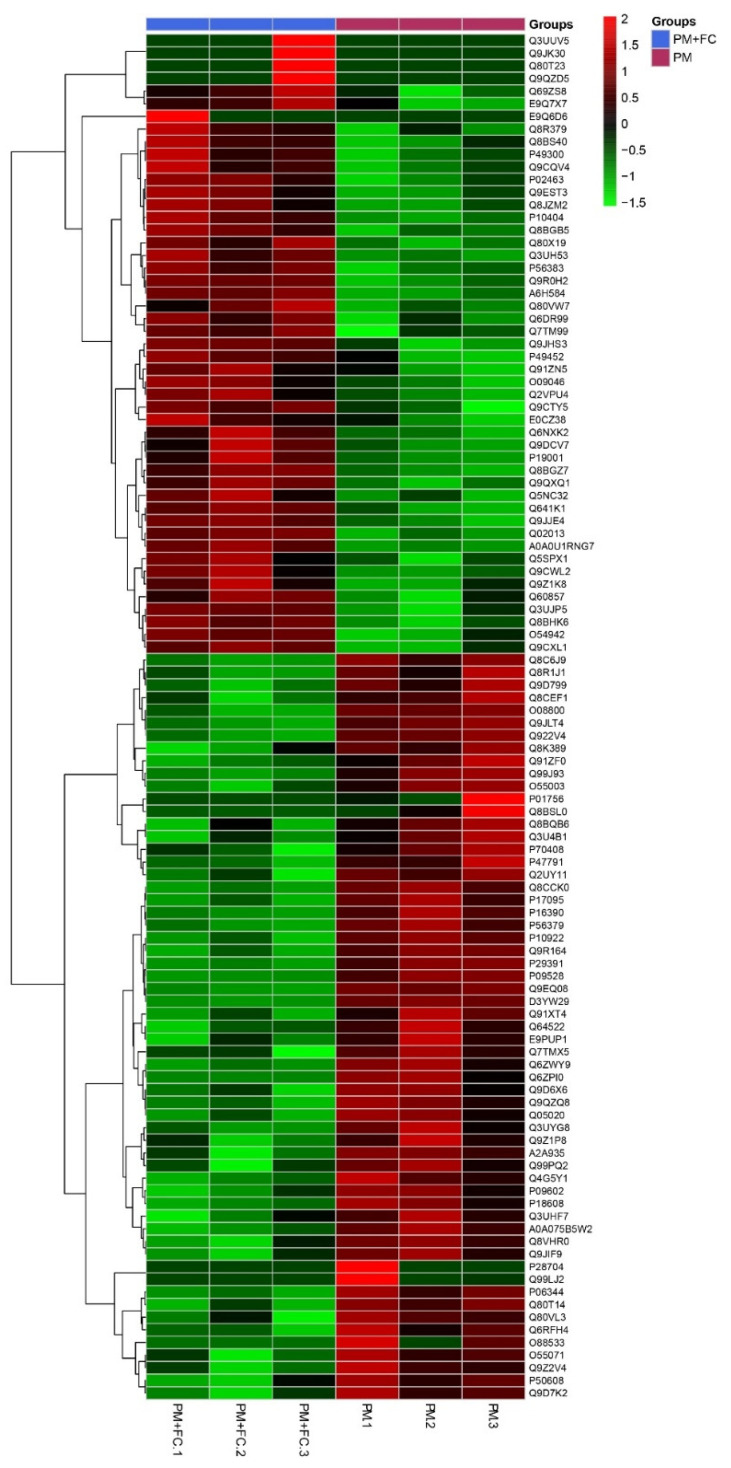
Hierarchical cluster analysis (HCA) of different protein expressions in mouse lung tissues between group PM + FC and group PM through the pheatmap package (https://CRAN.R-project.org/package=pheatmap assessed on 6 June 2021).

**Figure 7:**
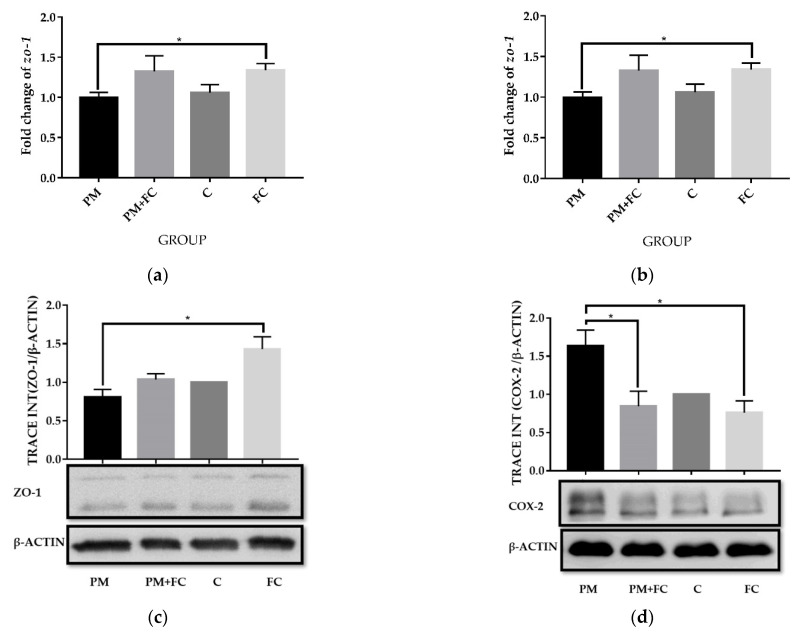
The expression levels of ZO-1 and COX-2 in mouse lung tissues: (**a**) mRNA expression levels of *zo-1*; (**b**) mRNA expression levels of *cox-2*; (**c**) the protein levels of ZO-1; (**d**) the protein levels of COX-2. * *p* < 0.05.

A total of 9875 proteins were identified among group C, group PM + FC, group FC and group PM. We compared the four groups in pairs and found the differential proteins according to the criteria of *p* < 0.05 (One-way ANOVA) and Unique Peptides ≥ 1, and the significant fold change was set at 1.3. The number of differential proteins were listed in Table S1. Hierarchical cluster analysis (HCA) is an algorithmic approach to find discrete groups with varying degrees of (dis)similarity in a data set represented by a (dis)similarity matrix. This analysis is processed with a pheatmap package (https://CRAN.R-project.org/package=pheatmap accessed on 6 June 2021). Figure S1 shows the HCA result, in which 59 proteins were upregulated and 49 were downregulated between group PM + FC and group PM using the cutoff value of 1.3-fold change (Figure 6). Both *zo-1* and *cox-2* signaling were found to have changed significantly (*p* < 0.05) when comparing group PM with group PM + FC using KEGG pathway analysis. We further observed that the *zo-1* mRNA levels of group FC were significantly higher than those of group PM (Figure 7a) and the *cox-2* mRNA expression levels of group FC were significantly lower than those of group C in mouse lung tissues (Figure 7b). Western blot showed similar results of ZO-1 (Figure 7c). Western blot also revealed that the levels of COX-2 in group FC and group PM + FC were lower than those in group PM (Figure 7d).

### 3.7. Effects of Herbal Extract Blend (FC) on Gut Microbiota

**Figure 8:**
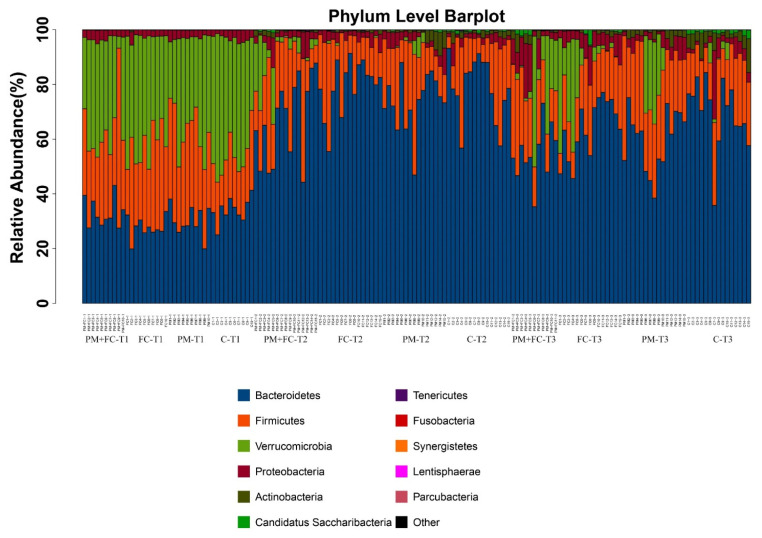
Phylum level barplots of mouse fecal microbiota of four groups (group PM, group PM + FC, group FC, and group C).

**Figure 9:**
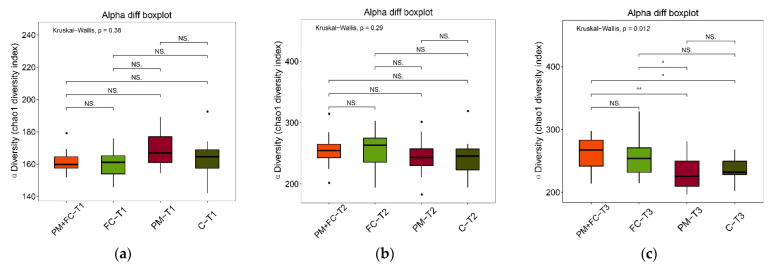
α diversity of gut microbiota at T1 (**a**), T2 (**b**), and T3 (**c**). NS *p* ≥ 0.05, * *p* < 0.05, ** *p* < 0.01.

**Figure 10:**
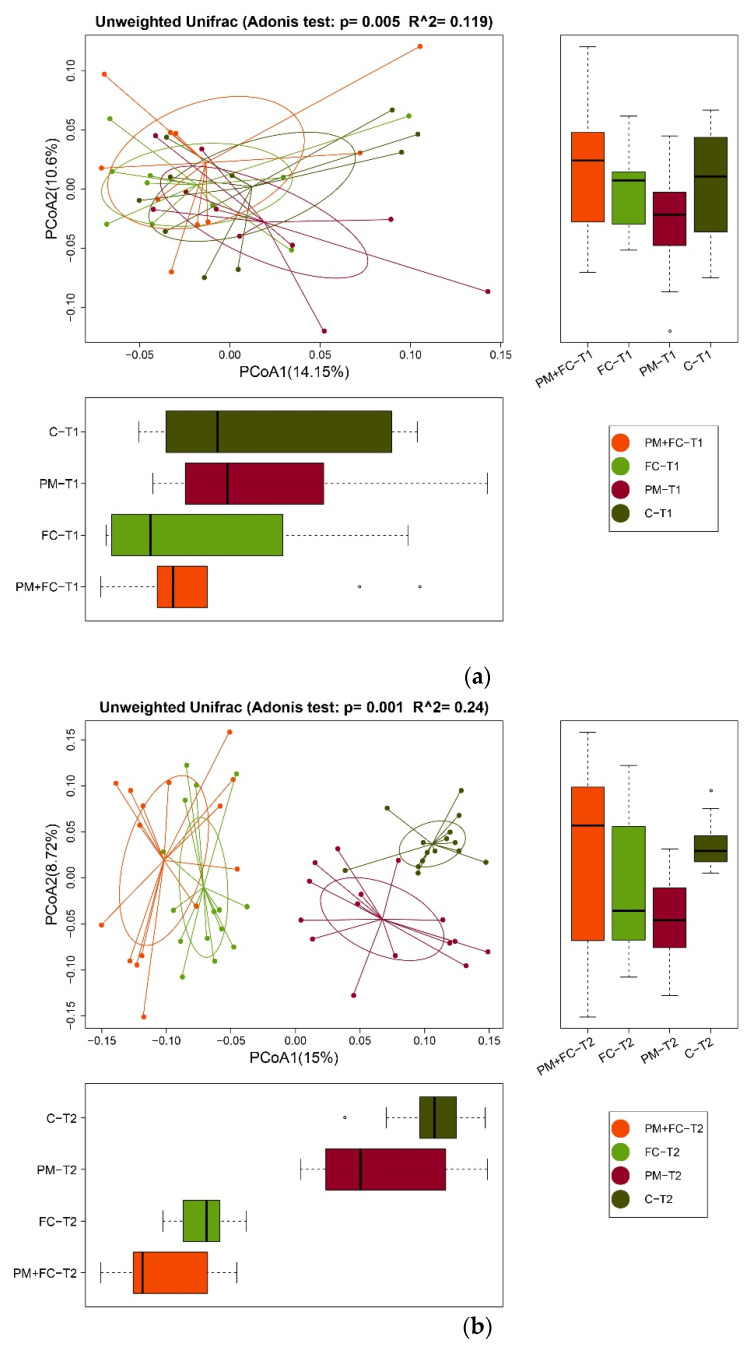

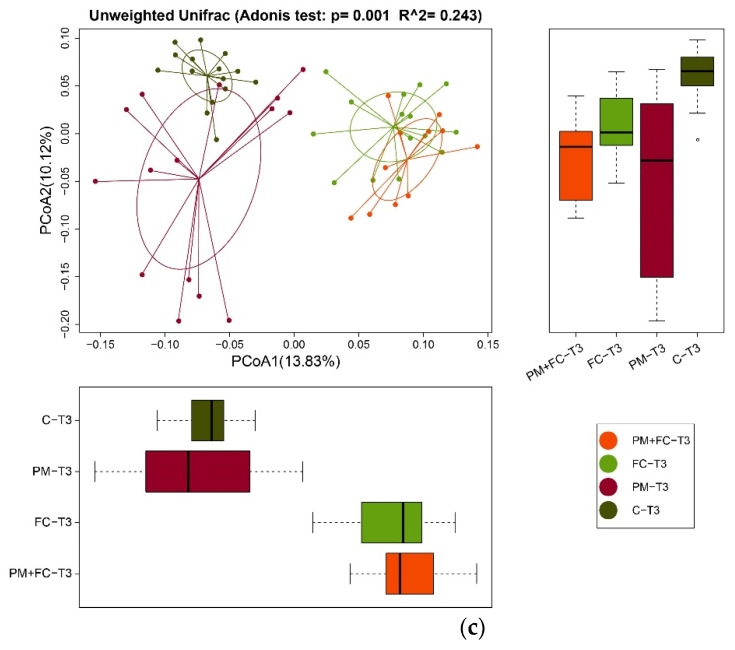
β diversity of gut microbiota at T1 (**a**), T2 (**b**), and T3 (**c**). The abscissa and ordinate represent the first and second principal coordinates, respectively; the percentage represents the contribution rate of the corresponding principal coordinate to the sample difference; and the *p* value is the test *p* value of the corresponding principal coordinate. The dots represent each sample, respectively, and different colors indicate that the samples belong to different groups. The horizontal box graph is the distribution of the values of different groups on the first principal coordinate; the vertical box graph is the distribution of the values of different groups on the second principal coordinate. The calculation process was implemented by the vegan package of the R language: 0.01 < *p* ≤ 0.05, indicating significant difference; *p* ≤ 0.01, indicating an extremely distinct difference.

**Figure 11:**
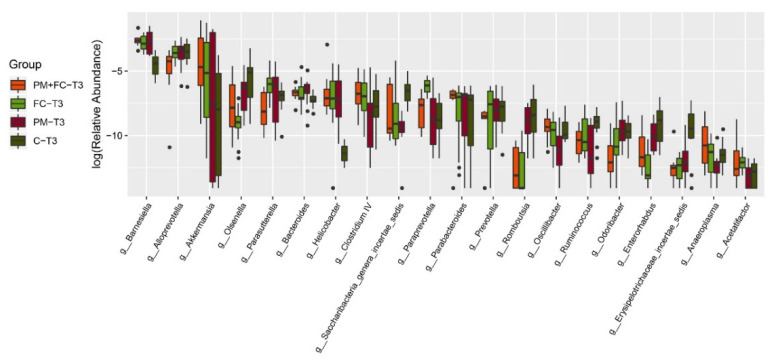
Abundance of significantly different bacteria in gut microbiota among four groups at T3.

**Figure 12:**
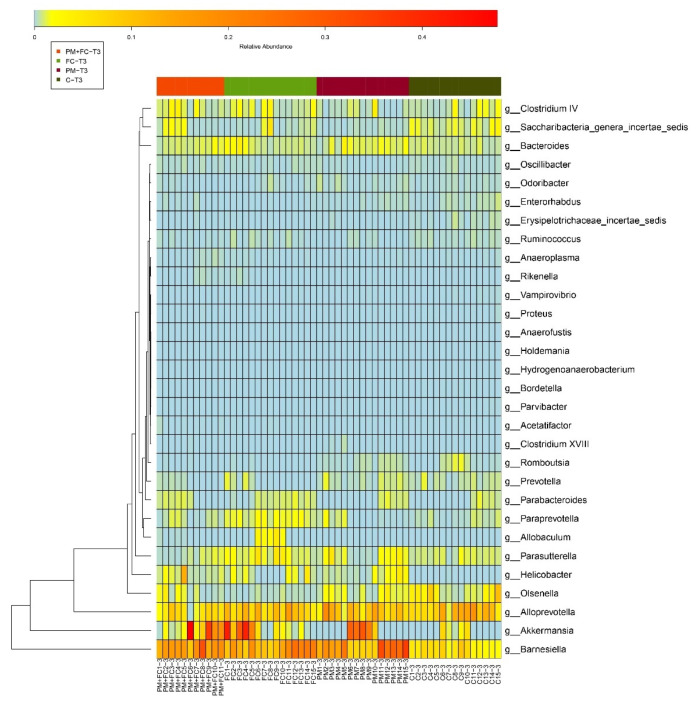
Heatmaps and clustering of individual samples for gut microbiota taxonomic composition.

The gut microbiota composition was analyzed for all groups by 16S rDNA sequencing. Figure 8 is a phylum level bar plot of all samples of the four groups at T1 (before FC gavage), T2 (before nasal drip experiment), and T3 (after nasal drip experiment). The gut microbiota of mice mainly covered the phyla of Bacteroidetes, Firmicutes, Verrucomicrobia, and Proteobacteria. At T2, the Verrucomicrobia was significantly reduced to the baseline, while the proportion of Bacteroidetes, Proteobacteria, and Actinobacteria was significantly increased. At T3, Verrucomicrobia increased in some samples. Compared with T1, the microbiota of T2 and T3 showed significant changes. α diversity provides a summary statistic of the microbial community, whereby higher α diversity indicates a greater number of species [46]. Low α diversity is considered an adverse but nonspecific characteristic [47]. We can see that α diversity of group PM decreased significantly at T3, while α diversity of group FC increased significantly compared with group C (Figure 9). β diversity, also known as between-habitat diversity, is a measure of interindividual (between samples) diversity that assesses similarity of communities compared with the other samples analyzed [46]. The β diversity of mouse microbiota showed similar results at T3 (Figure 10). Figure 11 lists the bacterial genera and shows significant differences among groups. Figure 12 shows the heatmap of the four groups at genus level. The relative abundance of the genera *Clostridium IV*, *Saccharibacteria_genera_incertae_sedis*, and *Helicobacter* increased, while *Olsenella*, *Barnesiella*, *Romboutsia*, *Odoribacter*, *Alistipes*, and *Bacteroides* decreased in group PM + FC compared with group PM. The relative abundance of the genera *Barnesiella, Helicobacter, Akkermansia, Allobaculum, Paraprevotella*, *Parasutterella,* and *Clostridium IV* increased while *Olsenella, Enterohabdus*, and *Romboutsia* decreased in group FC compared with group C. The genera *Barnesiella, Helicobacter*, *Akkermansia, Clostridium IV*, and *Bacteroides* increased, while *Olsenella and Romboutsia* decreased in group PM + FC compared with group C. The relative abundance of genera *Parabacteroides* and *Helicobacter* increased in group PM compared with group C.

## 4. Discussion

Herbal extracts are widely used in multifunctional products and play a role in health promotion based on their effectiveness and safety [48,49]. As mentioned above, FC is a formulation with three natural ingredients, including honeysuckle (Lonicera japonica) extract, monk fruit (Luo Han Guo) extract, and broccoli seed extract, with the active ingredients being chlorogenic acid, Mogrosides V, and glucoraphanin, respectively. All three of the natural ingredients in FC are widely used in food and have long been considered safe. However, since safety is the highest priority for food applications, it is helpful to further evaluate the safety of the blended product. According to the 2015 edition of ChP, the dose of clinical administrations of Lonicera japonica is suggested to be 6–15 g per day in an adult, indicating it to be in the category of low-toxicity herbs. In the United States, monk fruit is generally considered safe, while in Canada, it is classified as a medicinal ingredient and permitted as a flavor enhancer and sweetener [50]. Clinical trials applying glucoraphanin and sulforaphane to treat disease show that they do not affect body weight, organ weight, or hematological, clinical chemistry, pathological indicators, nor do they affect thyroid function or thyroid autoimmunity [51,52], indicating their safe use as food ingredients. In our research, this herbal extract blend (FC) demonstrated acceptable safety in cells in vitro, with the lowest toxic concentration being much higher than the dosage we applied.

All three natural ingredients, especially glucoraphanin, are considered to have superior anti-inflammatory properties. Lonicera japonica has been reported to effect the release of inflammatory factors (*tnf-α*, *il-1β*, *il-6* and *ifn-γ*) through several signaling pathways, such as the IL-17 signaling pathway and the p38/MAPK signaling pathway [53,54,55]. According to some studies, Mogroside V is also able to reduce the expression of inflammatory factors such as *il-1β*, *il-6*, and *tnf-α*, and to inhibit the activation of *cox-2* [26,56,57]. Glucoraphanin can be transformed into the active substance sulforaphane under the action of human intestinal flora or myrosinase, and sulforaphane can further activate the Nrf2 signaling pathway of human cells to exert an anti-inflammatory effect [58,59,60]. There has been little research studying the activity of the combination of these three components, especially the function of glucoraphanin in respiratory tract irritation or inflammation. Our research demonstrated results similar to those of previous studies. Cell experiments showed that the levels of pro-inflammatory factors (*il-1β, il-6, il-8,* and *tnf-α*) in group FC and group PM + FC were significantly lower than those in group PM, and the level of *cox-2* in group FC was also significantly inhibited compared to group PM. COX-2 is an inducible enzyme triggered by a number of cytokines and inflammatory mediators present in various inflammatory cells [61]. Recent findings demonstrated that COX-2–dependent regulation of TGF-β and IL-6 is involved in the differentiation of naive CD41 T cells to Th17 cells [62]. In our results, the expression of COX-2 was induced by PM2.5 toward a pro-inflammation direction, while FC presented an anti-inflammatory effect through downregulated COX-2 expression. Animal experiments also showed higher levels of *il-10* expression and lower levels of *cox-2* expression in group FC and group PM + FC, indicating that FC has a remarkable anti-inflammatory effect and could be used as functional food to protect the gut and lung from inflammation. However, since PM2.5 is not a strong irritant, our short-term acute model did not induce typical pathological inflammation, and the increase in the expression of inflammatory factors was not significant, showing a low-grade inflammatory state. Nevertheless, FC can still play a role in maintaining the homeostasis of the respiratory tract and the gut. However, the anti-inflammatory effects of FC displayed different mechanisms in the lung and the gut. The gut is believed to be one of the most important immune organs in the body; the inflammatory disorder in the gut is common and difficult to cure. FC can directly reach the intestinal tract through oral administration, so the protective effect is more important in the digestive system. For the lung, our results supported beneficial function of FC, but the mechanism needs to be further studied, since normally administered FC does not directly enter the respiratory tract.

The main ingredients of FC, Lonicera japonica, Momordica grosvenori, and broccoli, have not previously been widely studied in the context of GI microbiota [63]. The results of our study showed a significant impact of FC in regulating gut microbiota. FC increased both α diversity and β diversity of gut microbiota. In addition, FC positively impacted the composition of gut microbiota altered by PM exposure. The genera *Barnesiella* and *Akkermansia* were both significantly increased in group FC. Previous studies found that the presence of *Barnesiella* correlated with beneficial effects on human gut and was detected frequently in the feces of healthy people of various ages [64]. Moreover, cross-study analysis showed that *Barnesiella* genera were negatively correlated with common human intestinal diseases, such as Crohn’s disease, ulcerative colitis, colorectal cancer, and *Clostridioides difficile* infections [65]. *Akkermansia muciniphila* is an abundant resident of the intestinal tract that has received considerable attention in recent years as one of “next-generation of probiotics”. *A. muciniphila* has been shown to positively impact immune regulation and gut health protection. Both live and heat-killed *A. muciniphila* supernatant could stimulate the production of both anti- and proinflammatory cytokines (IL-1β, IL-6, IL-8, IL-10, and TNF-α), with the latter four induced at the highest levels [66]. Furthermore, *A. muciniphila*-derived extracellular vesicles (AmEVs) can activate AMPK, which is essential for the assembly of tight junction proteins at cell–cell junctions [67,68]. Oral administration of *A. muciniphila* can normalize the mucus thickness of the inner layer, increase the number of goblet cells, and upregulate the expression of tight-junction proteins, including occludin, claudins, and ZO-1, ZO-2, and ZO-3 in the gut of both HFD-feeding obese mice and mice with alcoholic fatty liver [69,70]. A study demonstrated that the intake of fermented and unfermented Flos Lonicera could also result in an increase of *A. muciniphila* abundance in the gut, which is consistent with the results in our study [63]. Accordingly, FC may perform an anti-inflammatory function in the intestine, enhance intestinal health, and produce other health benefits through favoring beneficial bacteria such as *Barnesiella* and *Akkermansia*. 

It has been reported that an elevated abundance of *A. muciniphila* in gut microbiota may improve metabolism in diabetes [70,71,72]. Moreover, Momordica grosvenori extract has been used as an alternative to sugar to reduce obesity in some studies [73,74]. Therefore, in addition to its anti-inflammatory effect, due to the inclusion of Momordica grosvenori extract and the upregulation of *A. muciniphila*, FC may play a role as a diabetic food supplement. This concept is worthy of future validation. All the studies in which animals were treated with *A.* muciniphila showed that the bacteria lowered body weight and fat-mass gain [75]. In our study, the weight of the mice in group FC was significantly lower than that of group C from the 13th week of the experiment. We believe that the application of FC may have a weight control effect. However, in the 15th and 16th weeks, no significant differences were observed among different groups. It is possible that nasal instillation or the PM2.5 factor added in the 15th and 16th weeks affected the weight monitoring. Further research should be carried out in regard to this concept of weight control. 

Furthermore, since lung microbiota may also play a role in maintaining pulmonary homeostasis, this area may also be of great value for future study. Increasing evidence indicates the intimate relationship between the gastrointestinal tract and the respiratory tract. Exacerbations of chronic gut and lung disease have been shown to share key conceptual features with the disorder and dysregulation of the microbial ecosystem. Although the lung microbiota study is challenging, the modulation of lung microbiota by FC may be an important focus for future research.

## 5. Conclusions

FC herbal extracts were shown to relieve inflammation and affect the expression of tight junction proteins related to the integrity of gut epithelium in both cell and animal experiments. We also showed that FC may play a role in regulating gut microbiota, including increasing the abundance of beneficial bacteria. This protective effect may act, in part, through regulation of COX-2 expression. This study elucidated a protective role in inflammatory disease of a novel herbal extract that may be screened as a candidate for functional foods.

## Figures and Tables

**Table 1:** Primers used for quantitative real-time PCR.

Gene	Species		Sequences (5′-3′)
*Gapdh*	human	forward	CTGACTTCAACAGCGACACC
		reverse	TGCTGTAGCCAAATTCGTTGT
*il-1β*	human	forward	GAAATGCCACCTTTTGACAGTG
		reverse	TGGATGCTCATCAGGACAT
*il-6*	human	forward	CCGGAGAGGAGACTTCACAG
		reverse	CAGAATTGCCATTGCACA
*il-8*	human	forward	GACCACACTGCGCCAACAC
		reverse	CTTCTCCACAACCCTCTGCAC
*tnf-α*	human	forward	GAGGCCAAGCCCTGGTATG
		reverse	CGGGCCGATTGATCTCAGC
*ifn-γ*	human	forward	TCGGTAACTGACTTGAATGTCCA
		reverse	TCGCTTCCCTGTTTTAGCTGC
*claudin-1*	human	forward	TCTGGCTATTTTAGTTGCCACAG
		reverse	AGAGAGCCTGACCAAATTCGT
*zo-1*	human	forward	CCCCACTCTGAAAATGAGGA
		reverse	GGGAACAACATACAGTGACGC
*cox-2*	human	forward	GATACTCAGGCAGAGATGATCTACCC
		reverse	AGACCAGGCACCAGACCAAAGA
*β-actin*	mouse	forward	AGTGTGACGTTGACATCCGT
		reverse	TGCTAGGAGCCAGAGCAGTA
*il-10*	mouse	forward	CTTACTGACTGGCATGAGGATCA
		reverse	GCAGCTCTAGGAGCATGTGG
*tnf-α*	mouse	forward	CCTCCAGAAAAGACACCA
		reverse	ACAAGCAGGAATGAGAAGAG
*zo-1*	mouse	forward	TGAACGCTCTCATAAGCTTCGTAA
		reverse	ACCGTACCAACCATCATTCATTG
*cox-2*	mouse	forward	GAAGTCTTTGGTCTGGTGCCTG
		reverse	GTCTGCTGGTTTGGAATAGTTGC

**Table 2:** The chemical composition of PM2.5 collected in Shanghai.

Parameters	Concentration (mg/kg)	Concentration (μg/m³)	Reference Value (Annual Average) (μg/m³)
Copper	43.7	1.71 × 10^−3^	
Nickel	13.3	5.21 × 10^−4^	
Zinc	615	2.41 × 10^−2^	
Vanadium	0.417	1.63 × 10^−5^	
Stibium	3	1.17 × 10^−4^	
Iron	4607	1.80 × 10^−1^	
Sulfide	1.02	3.99 × 10^−5^	60 (sulfur dioxide)
Naphthalin	0	0	
Acenaphthylene	0	0	
Acenaphthene	0	0	
Fluorene	0	0	
Phenanthrene	0	0	
Anthracene	0	0	
Fluoranthene	0.013	5.09 × 10^−7^	
Pyrene	0.011	4.31 × 10^−7^	
Benzo(a)anthracene	0.007	2.74 × 10^−7^	
Chrysene	0.01	3.92 × 10^−7^	
Benzo(b)fluoranthene	0.022	8.61 × 10^−7^	
Benzo(k)fluoranthene	0.011	4.31 × 10^−7^	
Benzo(a)pyrene	0.006	2.35 × 10^−7^	0.001
Indene(1,2,3-cd)pyrene	0.017	6.66 × 10^−7^	
Dibenzo(a,h)anthracene	0.01	3.92 × 10^−7^	
Benzo(ghi)perylene	0.02	7.83 × 10^−7^	

## Data Availability

Data are available from the corresponding author on reasonable request.

## Supplementary Materials

The following supporting information can be downloaded at: https://www.mdpi.com/article/10.3390/nu14102010/s1.

## References

[1] Hsu C.-Y., Chiang H.-C., Lin S.-L., Chen M.-J., Lin T.-Y., Chen Y.-C. (2016). Elemental characterization and source apportionment of PM 10 and PM 2.5 in the western coastal area of central Taiwan. Sci. Total Environ..

[2] Chen R., Yin P., Meng X., Liu C., Wang L., Xu X., Ross J.A., Tse L.A., Zhao Z., Kan H. (2017). Fine Particulate Air Pollution and Daily Mortality. A Nationwide Analysis in 272 Chinese Cities. Am. J. Respir. Crit. Care Med..

[3] Gripenback S., Lundgren L., Eklund A., Liden C., Skare L., Tornling G., Grunewald J. (2005). Accumulation of eosinophils and T-lymphocytes in the lungs after exposure to pinewood dust. Eur. Respir. J..

[4] Salim S.Y., Kaplan G.G., Madsen K.L. (2014). Air pollution effects on the gut microbiota: A link between exposure and inflammatory disease. Gut Microbes.

[5] He M., Ichinose T., Yoshida S., Nishikawa M., Mori I., Yanagisawa R., Takano H., Inoue K., Sun G., Shibamoto T. (2010). Urban particulate matter in Beijing, China, enhances allergen-induced murine lung eosinophilia. Inhal. Toxicol..

[6] Park E.J., Roh J., Kim Y., Park K., Kim D.S., Yu S.D. (2011). PM 2.5 collected in a residential area induced Th1-type inflammatory responses with oxidative stress in mice. Environ. Res..

[7] Yoshizaki K., Brito J.M., Toledo A.C., Nakagawa N.K., Piccin V.S., Junqueira M.S., Negri E.M., Carvalho A.L., Oliveira A.P., Lima W.T. (2010). Subchronic effects of nasally instilled diesel exhaust particulates on the nasal and airway epithelia in mice. Inhal. Toxicol..

[8] Fu H., Liu X., Li W., Zu Y., Zhou F., Shou Q., Ding Z. (2020). PM2.5 Exposure Induces Inflammatory Response in Macrophages via the TLR4/COX-2/NF-kappaB Pathway. Inflammation.

[9] Desai S.J., Prickril B., Rasooly A. (2018). Mechanisms of Phytonutrient Modulation of Cyclooxygenase-2 (COX-2) and Inflammation Related to Cancer. Nutr. Cancer.

[10] Simon L.S. (1999). Role and regulation of cyclooxygenase-2 during inflammation. Am. J. Med..

[11] Mutlu E.A., Comba I.Y., Cho T., Engen P.A., Yazici C., Soberanes S., Hamanaka R.B., Nigdelioglu R., Meliton A.Y., Ghio A.J. (2018). Inhalational exposure to particulate matter air pollution alters the composition of the gut microbiome. Environ. Pollut..

[12] Mutlu E.A., Engen P.A., Soberanes S., Urich D., Forsyth C.B., Nigdelioglu R., Chiarella S.E., Radigan K.A., Gonzalez A., Jakate S. (2011). Particulate matter air pollution causes oxidant-mediated increase in gut permeability in mice. Part. Fibre Toxicol..

[13] Tornavaca O., Chia M., Dufton N., Almagro L.O., Conway D.E., Randi A.M., Schwartz M.A., Matter K., Balda M.S. (2015). ZO-1 controls endothelial adherens junctions, cell–cell tension, angiogenesis, and barrier formation. J. Cell Biol..

[14] Odenwald M.A., Choi W., Buckley A., Shashikanth N., Joseph N.E., Wang Y., Warren M.H., Buschmann M.M., Pavlyuk R., Hildebrand J. (2017). ZO-1 interactions with F-actin and occludin direct epithelial polarization and single lumen specification in 3D culture. J. Cell Sci..

[15] He X., Zhang L., Hu L., Liu S., Xiong A., Wang J., Xiong Y., Li G. (2021). PM2.5 Aggravated OVA-Induced Epithelial Tight Junction Disruption Through Fas Associated via Death Domain-Dependent Apoptosis in Asthmatic Mice. J. Asthma Allergy.

[16] Kuo W.T., Zuo L., Odenwald M.A., Madha S., Singh G., Gurniak C.B., Abraham C., Turner J.R. (2021). The Tight Junction Protein ZO-1 Is Dispensable for Barrier Function but Critical for Effective Mucosal Repair. Gastroenterology.

[17] Ananthakrishnan A.N., McGinley E.L., Binion D.G., Saeian K. (2011). Ambient air pollution correlates with hospitalizations for inflammatory bowel disease: An ecologic analysis. Inflamm. Bowel Dis..

[18] Wang W., Zhou J., Chen M., Huang X., Xie X., Li W., Cao Q., Kan H., Xu Y., Ying Z. (2018). Exposure to concentrated ambient PM2.5 alters the composition of gut microbiota in a murine model. Part. Fibre Toxicol..

[19] Huxley E.J., Viroslav J., Gray W.R., Pierce A.K. (1978). Pharyngeal aspiration in normal adults and patients with depressed consciousness. Am. J. Med..

[20] Li J., Hu Y., Liu L., Wang Q., Zeng J., Chen C. (2020). PM2.5 exposure perturbs lung microbiome and its metabolic profile in mice. Sci. Total Environ..

[21] Hilty M., Burke C., Pedro H., Cardenas P., Bush A., Bossley C., Davies J., Ervine A., Poulter L., Pachter L. (2010). Disordered microbial communities in asthmatic airways. PLoS ONE.

[22] Sze M.A., Hogg J.C., Sin D.D. (2014). Bacterial microbiome of lungs in COPD. Int. J. Chron. Obstruct. Pulmon. Dis..

[23] Lipuma J.J. (2010). The changing microbial epidemiology in cystic fibrosis. Clin. Microbiol. Rev..

[24] Mao Q., Jiang F., Yin R., Wang J., Xia W., Dong G., Ma W., Yang Y., Xu L., Hu J. (2018). Interplay between the lung microbiome and lung cancer. Cancer Lett..

[25] Nikzad-Langerodi R., Ortmann S., Pferschy-Wenzig E.M., Bochkov V., Zhao Y.M., Miao J.H., Saukel J., Ladurner A., Heiss E.H., Dirsch V.M. (2017). Assessment of anti-inflammatory properties of extracts from Honeysuckle (*Lonicera* sp. L., Caprifoliaceae) by ATR-FTIR spectroscopy. Talanta.

[26] Li Y., Zou L., Li T., Lai D., Wu Y., Qin S. (2019). Mogroside V inhibits LPS-induced COX-2 expression/ROS production and overexpression of HO-1 by blocking phosphorylation of AKT1 in RAW264.7 cells. Acta Biochim. Biophys. Sin..

[27] Kong D., Li Y., Bai M., Deng Y., Liang G., Wu H. (2017). A comparative study of the dynamic accumulation of polyphenol components and the changes in their antioxidant activities in diploid and tetraploid Lonicera japonica. Plant Physiol. Biochem..

[28] Xu Q., Chen S.Y., Deng L.D., Feng L.P., Huang L.Z., Yu R.R. (2013). Antioxidant effect of mogrosides against oxidative stress induced by palmitic acid in mouse insulinoma NIT-1 cells. Braz. J. Med. Biol. Res..

[29] Riedl M.A., Saxon A., Diaz-Sanchez D. (2009). Oral sulforaphane increases Phase II antioxidant enzymes in the human upper airway. Clin. Immunol..

[30] Park H.S., Park K.I., Lee D.H., Kang S.R., Nagappan A., Kim J.A., Kim E.H., Lee W.S., Shin S.C., Hah Y.S. (2012). Polyphenolic extract isolated from Korean Lonicera japonica Thunb. induce G2/M cell cycle arrest and apoptosis in HepG2 cells: Involvements of PI3K/Akt and MAPKs. Food Chem. Toxicol..

[31] Takasaki M., Konoshima T., Murata Y., Sugiura M., Nishino H., Tokuda H., Matsumoto K., Kasai R., Yamasaki K. (2003). Anticarcinogenic activity of natural sweeteners, cucurbitane glycosides, from Momordica grosvenori. Cancer Lett..

[32] Nandini D.B., Rao R.S., Deepak B.S., Reddy P.B. (2020). Sulforaphane in broccoli: The green chemoprevention!! Role in cancer prevention and therapy. J. Oral Maxillofac. Pathol..

[33] Kang O.H., Choi Y.A., Park H.J., Lee J.Y., Kim D.K., Choi S.C., Kim T.H., Nah Y.H., Yun K.J., Choi S.J. (2004). Inhibition of trypsin-induced mast cell activation by water fraction of *Lonicera japonica*. Arch. Pharm. Res..

[34] Muller L., Meyer M., Bauer R.N., Zhou H., Zhang H., Jones S., Robinette C., Noah T.L., Jaspers I. (2016). Effect of Broccoli Sprouts and Live Attenuated Influenza Virus on Peripheral Blood Natural Killer Cells: A Randomized, Double-Blind Study. PLoS ONE.

[35] Shi Z., Liu Z., Liu C., Wu M., Su H., Ma X., Zang Y., Wang J., Zhao Y., Xiao X. (2016). Spectrum-Effect Relationships Between Chemical Fingerprints and Antibacterial Effects of Lonicerae Japonicae Flos and Lonicerae Flos Base on UPLC and Microcalorimetry. Front. Pharmacol..

[36] Park J.W., Bae H., Lee G., Hong B.G., Yoo H.H., Lim S.J., Lee K., Kim J., Ryu B., Lee B.J. (2013). Prophylactic effects of Lonicera japonica extract on dextran sulphate sodium-induced colitis in a mouse model by the inhibition of the Th1/Th17 response. Br. J. Nutr..

[37] Paturi G., Mandimika T., Butts C.A., Zhu S., Roy N.C., McNabb W.C., Ansell J. (2012). Influence of dietary blueberry and broccoli on cecal microbiota activity and colon morphology in mdr1a(^−/−^) mice, a model of inflammatory bowel diseases. Nutrition.

[38] Tae J., Han S.-W., Yoo J.-Y., Kim J.-A., Kang O.-H., Baek O.-S., Lim J.-P., Kim D.-K., Kim Y.-H., Bae K.-H. (2003). Anti-inflammatory effect of Lonicera japonica in proteinase-activated receptor 2-mediated paw edema. Clin. Chim. Acta.

[39] Shang X., Pan H., Li M., Miao X., Ding H. (2011). Lonicera japonica Thunb.: Ethnopharmacology, phytochemistry and pharmacology of an important traditional Chinese medicine. J. Ethnopharmacol..

[40] Song J.L., Qian B., Pan C., Lv F., Wang H., Gao Y., Zhou Y. (2019). Protective activity of mogroside V against ovalbumin-induced experimental allergic asthma in Kunming mice. J. Food Biochem..

[41] Giard D.J., Aaronson S.A., Todaro G.J., Arnstein P., Kersey J.H., Dosik H., Parks W.P. (1973). In vitro cultivation of human tumors: Establishment of cell lines derived from a series of solid tumors. J. Natl. Cancer Inst..

[42] Chanput W., Mes J.J., Wichers H.J. (2014). THP-1 cell line: An in vitro cell model for immune modulation approach. Int. Immunopharmacol..

[43] Jia H., Liu Y., Guo D., He W., Zhao L., Xia S. (2021). PM2.5-induced pulmonary inflammation via activating of the NLRP3/caspase-1 signaling pathway. Environ. Toxicol..

[44] Pu X.J., Li J., Zhou Q.L., Pan W., Li Y.Q., Zhang Y., Wang J., Jiao Z. (2018). Rosiglitazone inhibits PM2.5-induced cytotoxicity in human lung epithelial A549 cells. Ann. Transl. Med..

[45] Wang Y., Zhong Y., Hou T., Liao J., Zhang C., Sun C., Wang G. (2019). PM2.5 induces EMT and promotes CSC properties by activating Notch pathway in vivo and vitro. Ecotoxicol. Environ. Saf..

[46] Simpson C.A., Diaz-Arteche C., Eliby D., Schwartz O.S., Simmons J.G., Cowan C.S.M. (2021). The gut microbiota in anxiety and depression—A systematic review. Clin. Psychol. Rev..

[47] Verhaar B.J.H., Prodan A., Nieuwdorp M., Muller M. (2020). Gut Microbiota in Hypertension and Atherosclerosis: A Review. Nutrients.

[48] Rehman S.U., Choe K., Yoo H.H. (2016). Review on a Traditional Herbal Medicine, Eurycoma longifolia Jack (Tongkat Ali): Its Traditional Uses, Chemistry, Evidence-Based Pharmacology and Toxicology. Molecules.

[49] Maleki-Saghooni N., Karimi F.Z., Behboodi Moghadam Z., Mirzaii Najmabadi K. (2018). The effectiveness and safety of Iranian herbal medicines for treatment of premenstrual syndrome: A systematic review. Avicenna J. Phytomed..

[50] Williamson E.M., Liu X., Izzo A.A. (2020). Trends in use, pharmacology, and clinical applications of emerging herbal nutraceuticals. Br. J. Pharmacol..

[51] Zhou Y., Yang H., Li Y., Lynch B., Jia X. (2015). Broccoli seed extract: Genotoxicity and subchronic toxicity studies. Regul. Toxicol. Pharmacol..

[52] Chartoumpekis D.V., Ziros P.G., Chen J.G., Groopman J.D., Kensler T.W., Sykiotis G.P. (2019). Broccoli sprout beverage is safe for thyroid hormonal and autoimmune status: Results of a 12-week randomized trial. Food Chem. Toxicol..

[53] Su X., Zhu Z.H., Zhang L., Wang Q., Xu M.M., Lu C., Zhu Y., Zeng J., Duan J.A., Zhao M. (2021). Anti-inflammatory property and functional substances of Lonicerae Japonicae Caulis. J. Ethnopharmacol..

[54] Liu C., Yin Z., Feng T., Zhang M., Zhou Z., Zhou Y. (2021). An integrated network pharmacology and RNA-Seq approach for exploring the preventive effect of Lonicerae japonicae flos on LPS-induced acute lung injury. J. Ethnopharmacol..

[55] Tzeng T.F., Liou S.S., Chang C.J., Liu I.M. (2014). The ethanol extract of *Lonicera japonica* (*Japanese honeysuckle*) attenuates diabetic nephropathy by inhibiting p-38 MAPK activity in streptozotocin-induced diabetic rats. Planta Med..

[56] Liu Y., Zhang B., Liu J., Qiao C., Xue N., Lv H., Li S. (2021). Mogroside V Alleviates Lipopolysaccharide-Induced Neuroinflammation via Inhibition of TLR4-MyD88 and Activation of AKT/AMPK-Nrf2 Signaling Pathway. Evid. Based Complement. Altern. Med..

[57] Shi D., Zheng M., Wang Y., Liu C., Chen S. (2014). Protective effects and mechanisms of mogroside V on LPS-induced acute lung injury in mice. Pharm. Biol..

[58] Schepici G., Bramanti P., Mazzon E. (2020). Efficacy of Sulforaphane in Neurodegenerative Diseases. Int. J. Mol. Sci..

[59] Uddin M.S., Mamun A.A., Jakaria M., Thangapandiyan S., Ahmad J., Rahman M.A., Mathew B., Abdel-Daim M.M., Aleya L. (2020). Emerging promise of sulforaphane-mediated Nrf2 signaling cascade against neurological disorders. Sci. Total Environ..

[60] Ruhee R.T., Suzuki K. (2020). The Integrative Role of Sulforaphane in Preventing Inflammation, Oxidative Stress and Fatigue: A Review of a Potential Protective Phytochemical. Antioxidants.

[61] Morita I. (2002). Distinct functions of COX-1 and COX-2. Prostaglandins Other Lipid Mediat..

[62] Carey M.A., Germolec D.R., Bradbury J.A., Gooch R.A., Moorman M.P., Flake G.P., Langenbach R., Zeldin D.C. (2003). Accentuated T helper type 2 airway response after allergen challenge in cyclooxygenase-1^−/−^ but not cyclooxygenase-2^−/−^ mice. Am. J. Respir. Crit. Care Med..

[63] Wang J.H., Bose S., Kim G.C., Hong S.U., Kim J.H., Kim J.E., Kim H. (2014). Flos Lonicera ameliorates obesity and associated endotoxemia in rats through modulation of gut permeability and intestinal microbiota. PLoS ONE.

[64] Kulagina E.V., Efimov B.A., Maximov P.Y., Kafarskaia L.I., Chaplin A.V., Shkoporov A.N. (2012). Species composition of Bacteroidales order bacteria in the feces of healthy people of various ages. Biosci. Biotechnol. Biochem..

[65] Mancabelli L., Milani C., Lugli G.A., Turroni F., Cocconi D., van Sinderen D., Ventura M. (2017). Identification of universal gut microbial biomarkers of common human intestinal diseases by meta-analysis. FEMS Microbiol. Ecol..

[66] Ottman N.A. (2015). Host Immunostimulation and Substrate Utilization of the Gut Symbiont Akkermansia muciniphila.

[67] Zhang L., Li J., Young L.H., Caplan M.J. (2006). AMP-activated protein kinase regulates the assembly of epithelial tight junctions. Proc. Natl. Acad. Sci. USA.

[68] Zheng B., Cantley L.C. (2007). Regulation of epithelial tight junction assembly and disassembly by AMP-activated protein kinase. Proc. Natl. Acad. Sci. USA.

[69] Grander C., Adolph T.E., Wieser V., Lowe P., Wrzosek L., Gyongyosi B., Ward D.V., Grabherr F., Gerner R.R., Pfister A. (2018). Recovery of ethanol-induced *Akkermansia muciniphila* depletion ameliorates alcoholic liver disease. Gut.

[70] Everard A., Belzer C., Geurts L., Ouwerkerk J.P., Druart C., Bindels L.B., Guiot Y., Derrien M., Muccioli G.G., Delzenne N.M. (2013). Cross-talk between *Akkermansia muciniphila* and intestinal epithelium controls diet-induced obesity. Proc. Natl. Acad. Sci. USA.

[71] Hasani A., Ebrahimzadeh S., Hemmati F., Khabbaz A., Hasani A., Gholizadeh P. (2021). The role of *Akkermansia muciniphila* in obesity, diabetes and atherosclerosis. J. Med. Microbiol..

[72] Depommier C., Everard A., Druart C., Plovier H., Van Hul M., Vieira-Silva S., Falony G., Raes J., Maiter D., Delzenne N.M. (2019). Supplementation with *Akkermansia muciniphila* in overweight and obese human volunteers: A proof-of-concept exploratory study. Nat. Med..

[73] Çicek S.S. (2020). Structure-Dependent Activity of Plant-Derived Sweeteners. Molecules.

[74] Soejarto D.D., Addo E.M., Kinghorn A.D. (2019). Highly sweet compounds of plant origin: From ethnobotanical observations to wide utilization. J. Ethnopharmacol..

[75] Cani P.D. (2018). Human gut microbiome: Hopes, threats and promises. Gut.

