# Supplementary material for: A Novel Herbal Extract Blend Product Prevents Particulate Matters-Induced Inflammation by Improving Gut Microbiota and Maintaining the Integrity of the Intestinal Barrier
Source: Nutrients. 2022 May 11;14(10):2010. doi: 10.3390/nu14102010 (PMC9145798; doi:10.3390/nu14102010)
Supplement: Supplementary file 1 [file nutrients-14-02010-s001.zip › nutrients-1664968-supplementary.pdf]

nutrients

MDPI

## Supplementary materials

**Table S1.** Differential lung proteins between groups with p < 0.05 (One way ANOVA), Unique Peptides ≥ 1, and the difference fold more than 1.3.

| Groups | Down-regulation | Up-regulation | Total |
|---|---|---|---|
| FC vs C | 8 | 44 | 52 |
| PM vs C | 17 | 39 | 56 |
| PM+FC vs C | 79 | 83 | 162 |
| PM+FC vs PM | 59 | 49 | 108 |

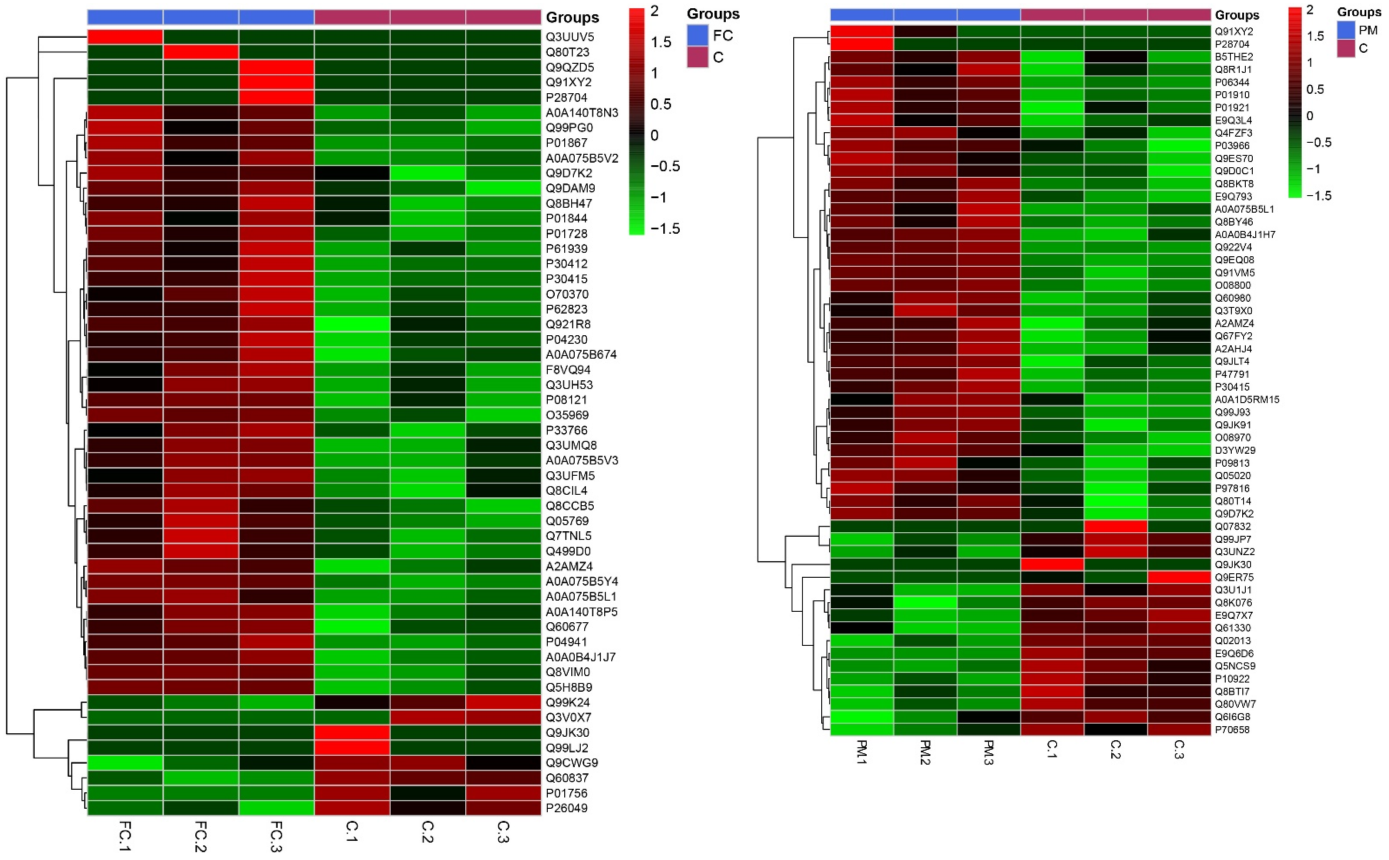


(a) (b)

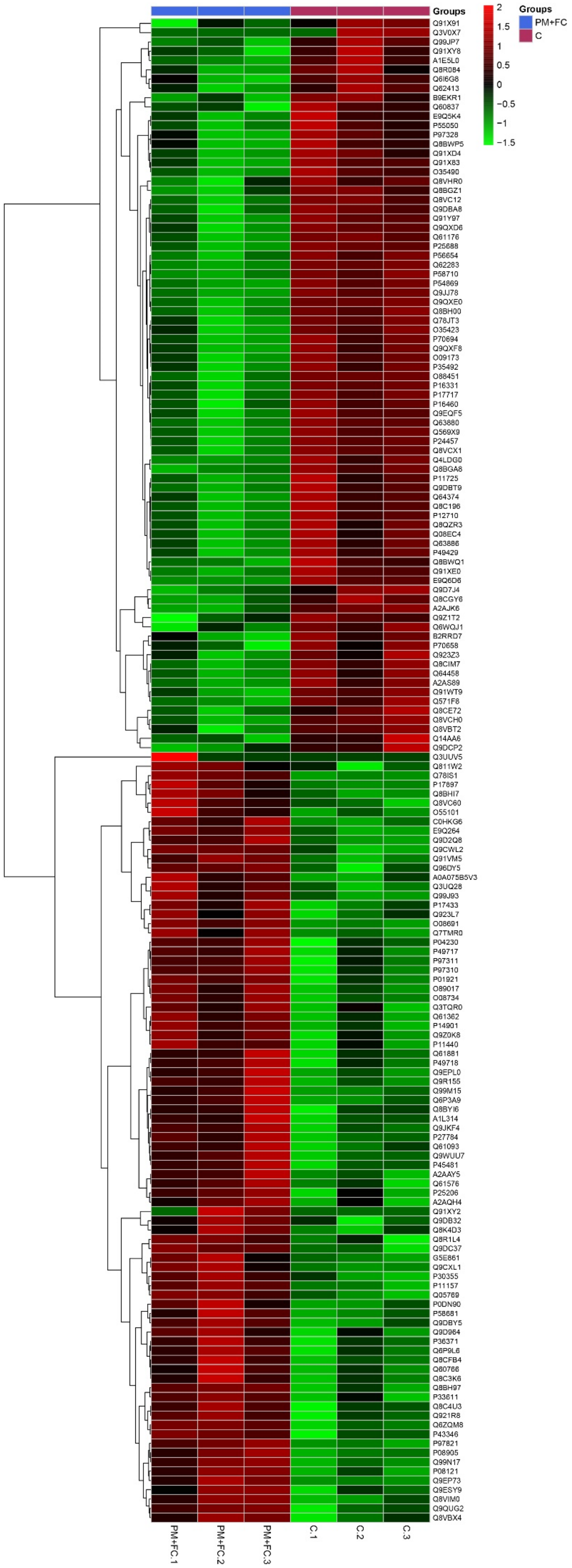
Groups
PM+FC
C
2
1.5
1
0.5
0
−0.5
−1
−1.5
PM+FC.1
PM+FC.2
PM+FC.3
C.1
C.2
C.3

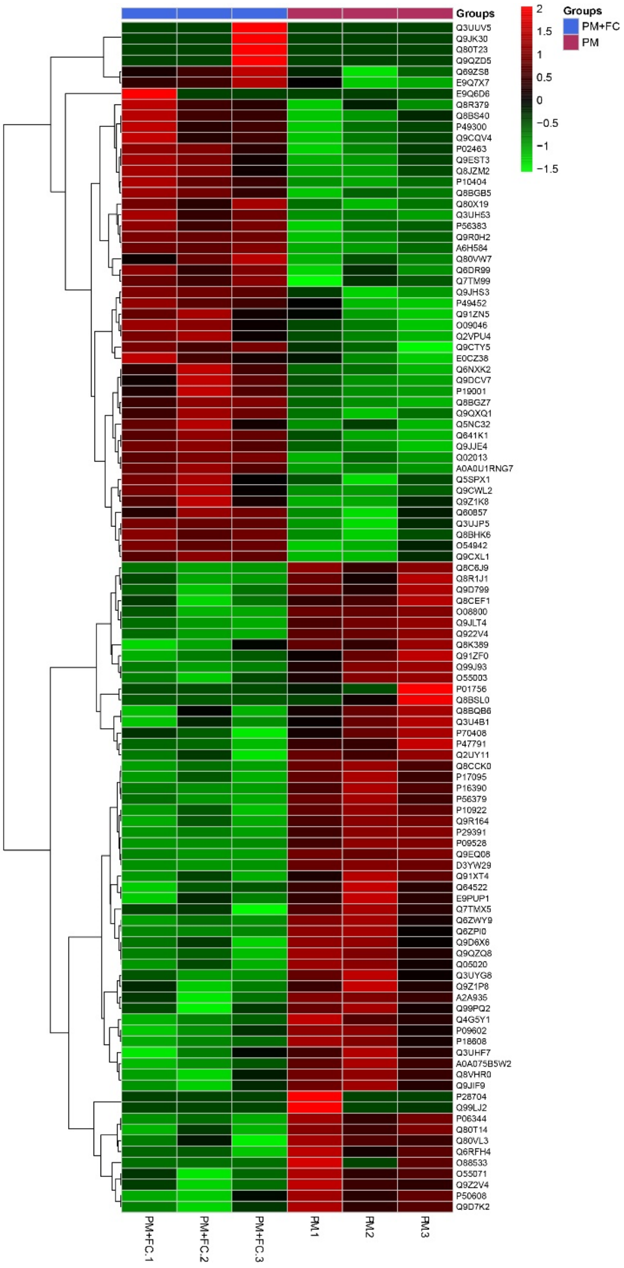
Groups
PM+FC
PM
2
1.5
1
0.5
0
−0.5
−1
−1.5
PM+FC.1
PM+FC.2
PM+FC.3
PM.1
PM.2
PM.3

(c) (d)

**Figure S1.** Hierarchical cluster analysis (HCA) of different protein expressions in mouse lung tissues between group FC and group C (a), between group PM and group C (b), between group PM+FC and group C (c), and between group PM+FC and group PM (d). .
